# Aberrant intestinal microbiota due to IL-1 receptor antagonist deficiency promotes IL-17- and TLR4-dependent arthritis

**DOI:** 10.1186/s40168-017-0278-2

**Published:** 2017-06-23

**Authors:** Rebecca Rogier, Thomas H. A. Ederveen, Jos Boekhorst, Harm Wopereis, Jose U. Scher, Julia Manasson, Sanne J. C. M. Frambach, Jan Knol, Johan Garssen, Peter M. van der Kraan, Marije I. Koenders, Wim B. van den Berg, Sacha A. F. T. van Hijum, Shahla Abdollahi-Roodsaz

**Affiliations:** 1 Experimental Rheumatology (272), Radboud University Medical Center, PO Box 9101, 6500HB Nijmegen, The Netherlands; 2 Centre for Molecular and Biomolecular Informatics, Radboud Institute for Molecular Life Sciences, Radboud University Medical Center, Nijmegen, The Netherlands; 3 NIZO food research, Ede, The Netherlands; 4 Danone Nutricia Research, Utrecht, The Netherlands; 5 Laboratory of Microbiology, Wageningen University, Wageningen, The Netherlands; 6 Division of Rheumatology, Department of Medicine, New York University School of Medicine, 301 East 17th Street, Room 1611A, New York, USA; 7 Division of Pharmacology, Utrecht Institute for Pharmaceutical Sciences, Utrecht University, Utrecht, The Netherlands

**Keywords:** Microbiota, Autoimmune arthritis, T helper 17 cells, Toll-like receptors, IL-1 receptor antagonist

## Abstract

**Background:**

Perturbation of commensal intestinal microbiota has been associated with several autoimmune diseases. Mice deficient in interleukin-1 receptor antagonist (*Il1rn* ^−/−^ mice) spontaneously develop autoimmune arthritis and are susceptible to other autoimmune diseases such as psoriasis, diabetes, and encephalomyelitis; however, the mechanisms of increased susceptibility to these autoimmune phenotypes are poorly understood. We investigated the role of interleukin-1 receptor antagonist (IL-1Ra) in regulation of commensal intestinal microbiota, and assessed the involvement of microbiota subsets and innate and adaptive mucosal immune responses that underlie the development of spontaneous arthritis in *Il1rn* ^*−*/*−*^ mice.

**Results:**

Using high-throughput 16S rRNA gene sequencing, we show that IL-1Ra critically maintains the diversity and regulates the composition of intestinal microbiota in mice. IL-1Ra deficiency reduced the intestinal microbial diversity and richness, and caused specific taxonomic alterations characterized by overrepresented *Helicobacter* and underrepresented *Ruminococcus* and *Prevotella*. Notably, the aberrant intestinal microbiota in *IL1rn* ^*−*/*−*^ mice specifically potentiated IL-17 production by intestinal lamina propria (LP) lymphocytes and skewed the LP T cell balance in favor of T helper 17 (Th17) cells, an effect transferable to WT mice by fecal microbiota. Importantly, LP Th17 cell expansion and the development of spontaneous autoimmune arthritis in *IL1rn* ^*−*/*−*^ mice were attenuated under germ-free condition. Selective antibiotic treatment revealed that tobramycin-induced alterations of commensal intestinal microbiota, i.e.*,* reduced *Helicobacter*, *Flexispira*, *Clostridium*, and *Dehalobacterium*, suppressed arthritis in *IL1rn* ^*−*/*−*^ mice. The arthritis phenotype in *IL1rn* ^*−*/*−*^ mice was previously shown to depend on Toll-like receptor 4 (TLR4). Using the ablation of both IL-1Ra and TLR4, we here show that the aberrations in the *IL1rn* ^*−*/*−*^ microbiota are partly TLR4-dependent. We further identify a role for TLR4 activation in the intestinal lamina propria production of IL-17 and cytokines involved in Th17 differentiation preceding the onset of arthritis.

**Conclusions:**

These findings identify a critical role for IL1Ra in maintaining the natural diversity and composition of intestinal microbiota, and suggest a role for TLR4 in mucosal Th17 cell induction associated with the development of autoimmune disease in mice.

**Electronic supplementary material:**

The online version of this article (doi:10.1186/s40168-017-0278-2) contains supplementary material, which is available to authorized users.

## Background

Interleukin-1 (IL-1) plays a central role in inflammation and immunity [1]. Activation of IL-1 receptor is physiologically controlled by its structural homologue and natural inhibitor, the IL-1 receptor antagonist (IL-1Ra), encoded by the *IL1rn* gene [2]. *IL1rn* knockout (*IL1rn* ^*−*/*−*^) mice are susceptible to a variety of autoimmune diseases including arthritis, psoriasis, diabetes, and encephalomyelitis [3–7]. This indicates a critical role for IL-1Ra in protection against autoimmunity; however, the mechanisms are poorly understood.

We questioned the role of IL-1Ra in regulation of the intestinal microbiota and the involvement of mucosal immune response as an underlying mechanism for the spontaneous autoimmune arthritis in *IL1rn* ^−/−^ mice, which is dependent on T cells and IL-17 [4, 8]. Several studies have associated commensal microbiota with autoimmune disease in mouse models of rheumatoid arthritis (RA), diabetes, and multiple sclerosis [9–13]. Importantly, the diversity and the composition of commensal intestinal microbiota are altered in patients with psoriatic and RA compared with healthy individuals [14–18]. One of the most prominent effects of microbiota is to define the balance between the proinflammatory CD4^+^ T helper 1 (Th1) and Th17 cells and protective regulatory T (Treg) cells, both at mucosal surfaces and systemically [19–21]. In this context, specific subsets of intestinal microbiota, such as the vancomycin-sensitive segmented filamentous bacteria (SFB), robustly induce differentiation of Th17 cells in small intestine LP (SI-LP) [22, 23]. Th17 cells are considered to play a pathogenic role in a subset of patients with RA by producing proinflammatory mediators, such as IL-17, and inducing osteoclastogenesis [24–28]. Interestingly, SFB colonization has been shown to exacerbate arthritis in K/BxN mice, an autoimmune model of arthritis arising from T cell auto-reactivity to the glycolytic enzyme glucose-6-phosphate isomerase [13, 29]. However, given that SFB were not found in human adults [30, 31], it is important to investigate the involvement of other indigenous microbiota in arthritis.

We previously described that arthritis in *IL1rn* ^−/−^ mice is diminished under germ-free (GF) condition [12]. We also showed that *IL1rn* ^−/−^ arthritis is dependent on the activation of Toll-like receptor 4 (TLR4), which affected systemic Th17 cell differentiation [12]. Here, we characterized the intestinal microbiota present in autoimmune-prone *IL1rn* ^−/−^ mice to clarify the nature of the microbiota that trigger arthritis and the underlying mucosal immune pathways. We also examined the role of TLR4 in the intestinal mucosal immune responses associated with arthritis.

Using high-throughput 16S ribosomal RNA (rRNA) gene sequencing of fecal microbiota, we demonstrate a critical role for IL-1Ra in maintaining the natural diversity and composition of commensal intestinal microbiota. We show that the aberrant *IL1rn* ^−/−^ microbiota increases intestinal Th17 cell differentiation, a phenotype that is transferable to wild-type (WT) mice by the microbiota. We also provide evidence that tobramycin-sensitive indigenous commensal intestinal bacteria contribute to arthritis in *IL1rn* ^−/−^ mice and identify a significant role for TLR4 in mucosal induction of IL-1β and IL-17 prior to the onset of arthritis.

## Results

### IL-1Ra maintains the biodiversity and richness of commensal intestinal microbiota

To identify intestinal microbiota associated with arthritis, we sequenced fecal bacterial 16S rRNA genes of *IL1rn* ^−/−^ and age- and gender-matched WT control mice. Fecal microbiota were analyzed as an unselected representation of the overall microbial communities in the intestines. Considering differential roles of TLR2 and TLR4 in *IL1rn* ^−/−^ arthritis [12], we sequenced samples of *IL1rn* ^−/−^ *Tlr2* ^−/−^ and *IL1rn* ^−/−^ *Tlr4* ^−/−^ mice in parallel. The average sequencing depth and total numbers of reads and operational taxonomic units (OTU) per experimental group as well as the hierarchical weighed UniFrac cluster analysis at the genus level are shown in Additional file 1: Table S1 and Additional file 1: Figure S1.

**Fig. 1:**
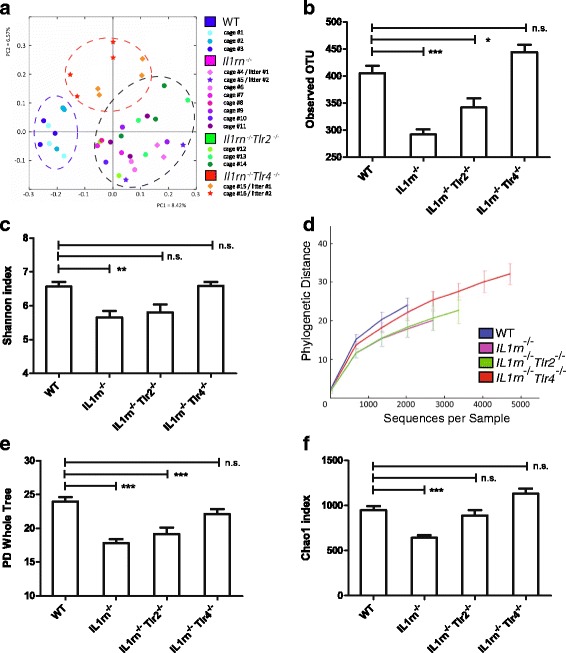
IL-1Ra deficiency skews intestinal microbial composition and reduces its diversity in a TLR4-dependent manner. **a** Principal coordinates analysis (PCoA) based on an unweighted UniFrac analysis of the intestinal microbial composition where samples of mice from different cages and litters are highlighted with different colors. The position and distance of data points indicates the degree of similarity in terms of both presence and relative abundance of bacterial taxonomies. **b** Number of observed operational taxonomic units (OTUs). **c** Shannon index of microbial diversity. **d** Alpha diversity rarefaction curves of phylogenetic distance (PD) whole tree. **e** PD whole tree, bootstrapped for 2000 reads per sample, averaged of four trials. **f** Chao index are shown. Data (mean + SEM) represent 16S rRNA gene 454-pyrosequencing analysis of intestinal microbiota of WT (*n* = 9), *IL1rn* ^−/−^ (*n* = 15), *IL1rn* ^−/−^ *Tlr2* ^−/−^ (*n* = 8), and *IL1rn* ^−/−^ *Tlr4* ^−/−^ (*n* = 8) mice. *n.s*. not significant,*P ≤ 0.05 and ***P ≤ 0.001, by Mann-Whitney *U* test. See also Additional file 1: Figure S1 and Additional file 1: Table S1

Principal coordinates analysis (PCoA) based on an unweighted UniFrac analysis of intestinal microbiota showed that *IL1rn* ^−/−^ microbiota is profoundly different from the WT microbiota (Fig. 1a). WT and *Il1rn* ^−/−^ mice formed clear, separate clusters regardless of the cage or litter of origin (Fig. 1a). Strikingly, microbial composition of *IL1rn* ^−/−^ and *IL1rn* ^−/−^ *Tlr2* ^−/−^ mice were indistinguishable, while *IL1rn* ^−/−^ *Tlr4* ^−/−^ mice formed another distinct cluster (Fig. 1a). To assess the effects of familial transmission and lineage origin versus the effect of the genotype (WT or *IL1rn* ^−/−^), we compared the UniFrac distances within a litter with UniFrac distances across litters of the same genotype as well as the opposite genotype, similar to the study by Ubeda et al. This analysis showed that the effect of the lineage origin and litter was limited in our experimental setting, because, as long as the genotype remained the same, the UniFrac distances across different litters were very similar to the UniFrac distances within the litters (Additional file 1: Figure S2A). This was true for both WT and *Il1rn* ^−/−^ groups. Importantly, the UniFrac distances were significantly higher when mice from different genotypes were compared, indicating a higher level of dissimilarity (Additional file 1: Figure S2B). Therefore, the effect of the genotype (*Il1rn*-deficiency) on the overall microbiota composition was significantly higher than any litter and cage effect. In addition, *IL1rn* ^−/−^ and *IL1rn* ^−/−^ *Tlr2* ^−/−^ mice showed significantly reduced number of OTUs and loss of microbial diversity based on the Shannon index, the rarefaction curves of phylogenetic distance (PD) whole tree, and the diversity index bootstrapped for the number of retrieved sequences (Fig. 1b–e). IL-1Ra deficiency also resulted in loss of species richness estimated by Chao index (Fig. 1f). These effects were fully or partially restored in *IL1rn* ^−/−^ *Tlr4* ^−/−^ mice (Fig. 1b–f). Altogether, these data strongly suggest that IL-1Ra plays a critical role in maintaining the intestinal microbial diversity, and that the loss of diversity in *IL1rn* ^−/−^ mice partially depends on TLR4.

### Specific taxonomic alterations characterize the dysregulated microbiota of autoimmune-prone IL1rn^−/−^ mice

**Fig. 2:**
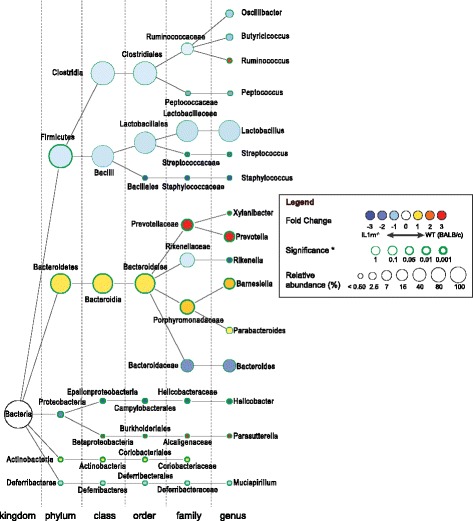
IL-1 receptor antagonist controls relative abundance of specific intestinal microbial taxa. Phylogenetic tree created by Cytoscape software showing specific changes in intestinal microbial community at different taxonomic levels induced by IL-1Ra deficiency. Nodes represent taxa, and the size of each node represents its relative abundance. The color *red* indicates a decrease and *blue* represents an increase of relative abundance in *IL1rn* ^−/−^ compared with WT mice. The thickness of the *green border* indicates the degree of the statistical significance by Mann-Whitney *U* test. See also Additional file 1: Table S2

The phylogenetic tree in Fig. 2 summarizes the observed alterations in relative abundances of microbial taxa. Compared with WT microbiota, we found a highly significant overrepresentation of the genus *Helicobacter* (*P* = 0.004, Bonferroni corrected), and a significant underrepresentation of the genus *Prevotella* (*P* = 0.008, Bonferroni corrected) (Fig. 2 and Additional file 1: Table S2). In addition, *IL1rn* ^−/−^ intestinal microbial composition was characterized by expansion of *Butyricimonas*, *Rikenella,* and *Streptococcus* by 10, 3.7, and 2.4 folds (*P* = 0.0048, *P* = 0.0022, and *P* = 0.0032, respectively, Bonferroni uncorrected), along with a decrease in *Parasutterella*, *Xylanibacter*, *Ruminococcus*, and *Barnesiella* by 10, 6.9, 2.7, and 1.4 folds (*P* = 0.040, *P* = 0.0004, *P* = 0.0099, and *P* = 0.0005, respectively, Bonferroni uncorrected), respectively (Fig. 2 and Additional file 1: Table S2)*.* Notably, we were unable to identify any OTUs in our dataset that could be classified as SFB (family *Clostridiaceae*, genus *Candidatus Arthromitus*). Moreover, none of the 27 present OTUs assigned to the family *Clostridiaceae* aligned with the known SFB 16S gene sequences in The Ribosomal Database Project [30]. However, SFB were detectable by qPCR in fecal samples of all WT mice and most of the *Il1rn* ^−/−^ mice (Additional file 1: Table S3). Although WT mice tended to have slightly more SFB, the level of SFB colonization was not significantly different between the groups (Additional file 1: Table S3).

Altogether, these data suggest that multiple yet specific microbial taxa are regulated by the physiologic expression of IL-1Ra. Therefore, a complex set of aberrant microbiota may affect the (mucosal) immune response and contribute to the autoimmune disease in *IL1rn* ^−/−^ mice.

### IL1rn^−/−^ intestinal microbiota potentiate IL-17 production by intestinal lamina propria lymphocytes

**Fig. 3:**
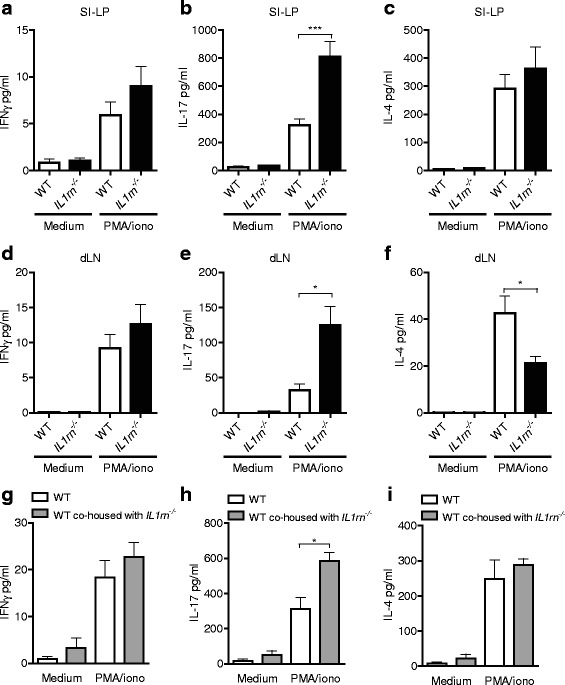
*Il1rn* ^−/−^ intestinal microbiota potentiate IL-17 production in intestinal lamina propria and joint-draining lymph nodes. **a**–**i** Production of prototypic Th1, Th17, and Th2 cell cytokines (IFNγ, IL-17A, and IL-4, respectively) by SI-LP (**a**–**c** and **g**–**i**) and draining lymph node (dLN) lymphocytes (**d**–**f**). Cells were isolated from WT and *IL1rn* ^−/−^ mice (**a**–**f**) or WT mice transplanted with *IL1rn* ^−/−^ feces and co-housed with *IL1rn* ^−/−^ mice for 10 days (**g**–**i**). Cells were stimulated ex vivo with PMA and ionomycin in duplicates for 5 h, and cytokines were measured by Luminex assay. Data represent mean + SEM of a representative experiment with *n* = 5 (**a–c**) and *n* = 3 (**d**–**i**) mice per group, each stimulated in duplicate. *n.s*. not significant, *P ≤ 0.05 and ***P ≤ 0.001, by Mann-Whitney *U* test. See also Additional file 1: Figure S2

To assess the effect of IL-1Ra deficiency on the mucosal T cell response, we cultured enzymatically isolated lamina propria lymphocytes (LPL) ex vivo in the presence of PMA and ionomycin. The production of the Th1 signature cytokine IFNγ was low and not altered by the IL-1Ra deficiency (Fig. 3a, gating strategy shown in Additional file 1: Figure S3); however, we observed a marked increase in the production of IL-17 by *IL1rn* ^−/−^ LPLs compared with WT LPLs (Fig. 3b). Flow cytometry analysis of lamina propria cells of WT and *IL1rn* ^−/−^ mice verified a significant, clear increase of IL-17-producing TCRβ^+^ CD4^+^ cells in *IL1rn* ^−/−^ mice, while TCRβ^−^ cells in LP produced similar amounts of IL-17 in WT and *IL1rn* ^−/−^ mice (Additional file 1: Figure S4). This suggests that Th17 cells, not γδ T cells, are the source of increased IL-17 production in LP of *IL1rn* ^−/−^ mice. Production of IL-4, IL-6, and TNFα was not affected (Fig. 3c and data not shown). Interestingly, the production of IL-17 but not IFNγ by lymphocytes in joint-draining lymph nodes (dLN) was significantly increased in *IL1rn* ^−/−^ mice compared with WT mice (Fig. 3d, e). This was paralleled by a concomitant decrease in the production of the Th2-related cytokine IL-4 in *IL1rn* ^−/−^ mice (Fig. 3f).

To identify a potential causative relationship between the aberrant microbiota and enhanced mucosal IL-17 production, we transferred *IL1rn* ^−/−^ microbiota to WT mice by oral gavage followed by immediate co-housing of the two mouse strains for up to 6 weeks. Transfer of *IL1rn* ^−/−^ microbiota clearly potentiated IL-17 production by SI-LP T cells in WT recipients as early as 10 days post fecal transfer and co-housing, without affecting IFNγ and IL-4 (Fig. 3g–i). This indicated that *IL1rn* ^−/−^ intestinal microbiota causes a shift in the LP T cell balance in favor of Th17 cells. However, this was not sufficient for the development of arthritis in WT animals during the 6-week follow-up period. This suggests that additional (genetic) susceptibility of the host, as in *IL1rn* ^−/−^ mice, is required for the development of arthritis. Furthermore, co-housing with WT mice did not affect the development of arthritis in *IL1rn* ^−/−^ mice (not shown).

### Potentiated Th17 response and spontaneous arthritis in IL1rn^−/−^ mice highly depend on the presence of commensal microbiota

**Fig. 4:**
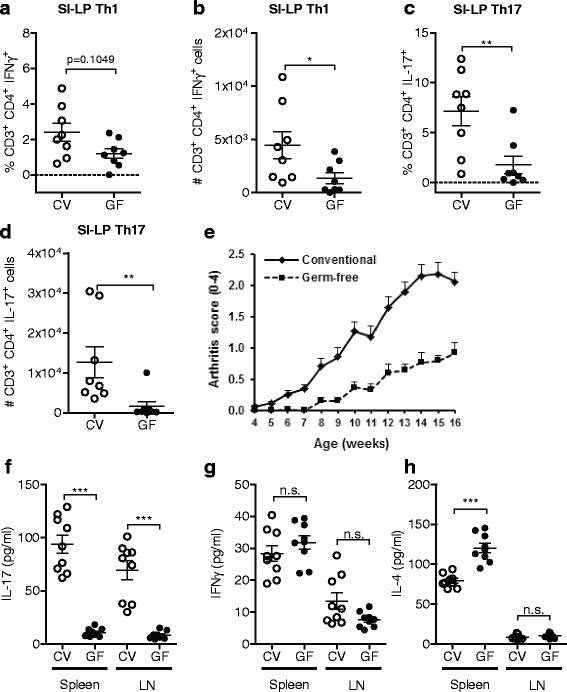
Commensal microbiota drive potentiated Th17 response and spontaneous arthritis in *IL1rn* ^−/−^ mice. **a**–**d** Frequency and numbers of IFNγ-producing (**a**, **b**) and IL-17-producing (**c**, **d**) CD3^+^CD4^+^ SI-LP cells. Data are pooled from three independent experiments. **e** Arthritis severity scores of conventional (CV, *n* = 12) and germ-free (GF, *n* = 11) *IL1rn* ^−/−^ mice of a representative experiment. Scale 0–2 for each hind paw. Mean + SEM is shown. **f**–**h** Production of IFNγ, IL-17, and IL-4 upon ex vivo stimulation of the spleen and lymph node cells from CV and GF mice with PMA and ionomycin for 6 h, as measured by Luminex assay. *n* = 3 mice per group of each stimulated in triplicate. *n.s*. not significant, **P ≤ 0.01 and ***P ≤ 0.001, by Mann-Whitney *U* test. See Additional file 1: Figure S3 for gating strategy

To determine whether the increase in intestinal Th17 cells and spontaneous arthritis in *IL1rn* ^−/−^ mice depends on commensal microbiota, we established GF *IL1rn* ^−/−^ mice. Flow cytometry analysis of LPLs showed that germ-free condition had no significant effect on the percentage of Th1 cells while reducing the numbers of Th1 cells in SI-LP (Fig. 4a, b). In contrast, both the percentage and the number of SI-LP Th17 cells were substantially reduced in GF compared with conventional (CV) mice (Fig. 4c, d). This strongly suggests that the skewed intestinal T cell balance in *IL1rn* ^−/−^ mice is largely microbiota dependent.

In agreement with IL-17-dependence of *IL1rn* ^−/−^ arthritis [4, 8] and in line with our previous observations [12], GF *IL1rn* ^−/−^ mice showed a clear sustained protection from arthritis with on average 3 weeks delay in disease onset (Fig. 4e). In addition, transfer of conventional *IL1rn* ^−/−^ microbiota to GF *IL1rn* ^−/−^ mice re-induced arthritis and resulted in a severe disease comparable to that in conventional *IL1rn* ^−/−^ mice (Additional file 1: Figure S5). Therefore, *IL1rn* ^−/−^ commensal microbiota, although not sufficient to induce arthritis in a WT host, are critical for the full development of arthritis in *IL1rn* ^−/−^ mice. Consistently, we also observed a robust reduction of IL-17, but not IFNγ, production in the spleen and most notably in the joint-adjacent lymph nodes of GF mice (Fig. 4f, g). These effects were accompanied by a significant reciprocal increase in Th2-related cytokines IL-4 and IL-10 as well as IL-2 in the spleens of GF mice (Fig. 4h and Additional file 1: Figure S6). These data support modulation of extra-intestinal immune response by intestinal microbiota during arthritis.

### Tobramycin-induced alteration of intestinal microbiota suppresses arthritis in IL1rn^−/−^ mice

**Fig. 5:**
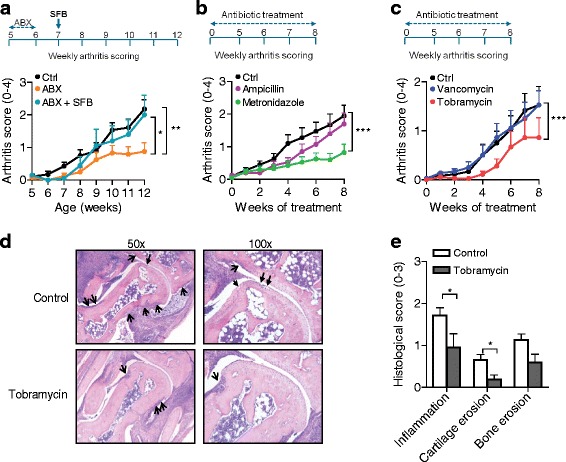
Commensal intestinal anaerobic tobramycin-sensitive microbiota promote arthritis in *IL1rn* ^−/−^ mice. **a** Arthritis severity scores (0–2 per paw) of *IL1rn* ^−/−^ mice treated with a cocktail of metronidazole, neomycin, and ampicillin (ABX) for 7 days (week 5 to 6), followed by re-colonization with SFB (ABX + SFB) 1 week after ending ABX treatment (at week 7). **b**, **c** Arthritis severity scores of *IL1rn* ^−/−^ mice either untreated (Ctrl) or treated with the mentioned antibiotic for 8 weeks. Data show mean + SEM of *n* = 5–7 (**a**) and *n* = 10 (**b**, **c**) mice per group. *P ≤ 0.05, ***P ≤ 0.001, by repeated measures ANOVA with Bonferroni correction for multiple testing. **d** Representative images of ankle joint sections of control and tobramycin-treated mice stained with hematoxylin and eosin illustrating decreased synovial inflammation, cartilage destruction (*closed arrows*), and bone erosion (*open arrows*). Original magnification ×50 (left panels) and ×100 (right panels). **e** Histopathologic scores (mean + SEM) of synovial inflammation, cartilage destruction, and bone erosion in control and tobramycin-treated *IL1rn* ^−/−^ mice. *n* = 9 per group. *P ≤ 0.05, by Mann-Whitney *U* test

The lack of microbiota in GF mice is not limited to the intestines. To determine whether intestinal microbiota serve as a relevant trigger for arthritis, we first depleted intestinal microbiota in conventionally housed mice using a cocktail of metronidazole, neomycin, and ampicillin. Treatment of 5-week-old *IL1rn* ^−/−^ mice for only 1 week suppressed arthritis over a sustained period, i.e., 6 weeks after ceasing antibiotics (Fig. 5a). This indicated that abrogation of arthritis in GF mice (Fig. 4c) is not due to an immature immune system and, more importantly, can be reproduced by the sole eradication of intestinal microbiota. Interestingly, colonization of the antibiotic-treated mice with SFB as model organisms inducing SI-LP Th17 cells was sufficient to fully restore arthritis (Fig. 5a).

To determine which subset of *IL1rn* ^−/−^ microbiota triggers arthritis, we first compared the effects of treatment with ampicillin, broadly targeting aerobic bacteria, and metronidazole, broadly targeting anaerobic bacteria. To our surprise, only metronidazole showed efficacy in reducing arthritis severity (Fig. 5b). This suggests involvement of anaerobic bacteria in the progression of arthritis. We next compared the effects of more selective antibiotics tobramycin and vancomycin, the latter of which has been reported to eradicate SFB and inhibit SFB-induced lamina propria Th17 cells and arthritis [13, 22, 32]. These experiments revealed that although SFB were able to exacerbate arthritis in *IL1rn* ^−/−^ mice (Fig. 5a), only tobramycin but not vancomycin significantly diminished arthritis (Fig. 5c). To understand the changes in the microbiota induced by tobramycin treatment, we compared 16S rRNA gene sequences of fecal microbiota at the end-point of tobramycin treatment with the baseline microbiota. Among taxa with >0.1% relative abundance, tobramycin treatment resulted in a near-complete elimination of the genera *Helicobacter* and *Flexispira* (both belonging to the family Helicobacteraceae). In addition, a strong and highly significant reduction in the genera *Clostridium* and *Dehalobacterium* was observed (Additional file 1: Figure S7 and Table S4). Other changes in the microbiota did not reach the statistical significance after Bonferroni correction for multiple testing. Therefore, tobramycin-induced alterations in these indigenous *IL1rn* ^−/−^ microbiota taxa resulted in suppression of arthritis. This was confirmed by histological examination of arthritic joints which showed a significant reduction of synovial inflammation as well as cartilage destruction and a non-significant reduction in bone erosion upon treatment with tobramycin (Fig. 5d, e).

### Aberrations of the intestinal microbiota and LP IL-17 production in IL1rn^−/−^ mice partly depend on TLR4

**Fig. 6:**
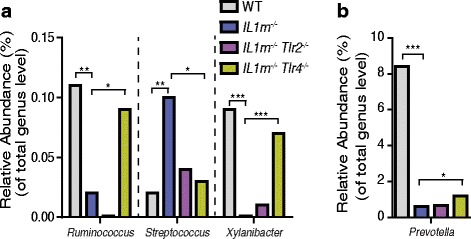
Alteration of specific intestinal microbiota in *IL1rn* ^−/−^ mice is partly TLR4 dependent. **a**, **b** Relative abundance of *Ruminococcus*, *Streptococcus*, *Xylanibacter*, and *Prevotella* in wild-type (WT) (*n* = 9), *IL1rn* ^−/−^ (*n* = 15), *IL1rn* ^−/−^ *Tlr2* ^−/−^ (*n* = 8), and *IL1rn* ^−/−^ *Tlr4* ^−/−^ (*n* = 8) mice. Data represent relative abundances of these genera obtained by 16S rRNA gene sequencing of the fecal microbiota

TLR4 plays a major role in recognition of Gram-negative bacteria [33]. We previously showed that *IL1rn* ^−/−^ *Tlr4* ^−/−^ mice have a marked and sustained reduction of arthritis [12]. Therefore, we assessed whether TLR4 plays a role in alterations of the intestinal microbiota and the induction of LP Th17 cells. A detailed analysis of the intestinal microbiota showed that in addition to the TLR4-dependent loss of microbial diversity in IL-1Ra-deficient mice (Fig. 1b–f), alterations in *Ruminococcus*, *Streptococcus*, and *Xylanibacter* were partially dependent on TLR4 and were restored in *IL1rn* ^−/−^ *Tlr4* ^−/−^ mice (Fig. 6a). Abundance of *Prevotella* was also restored to a statistically significant, yet minor extent (Fig. 6b). In total, 11 out of 44 taxa significantly altered in *IL1rn* ^−/−^ mice were normalized toward the WT levels in *IL1rn* ^−/−^ *Tlr4* ^−/−^ mice (Additional file 1: Table S2).

**Fig. 7:**
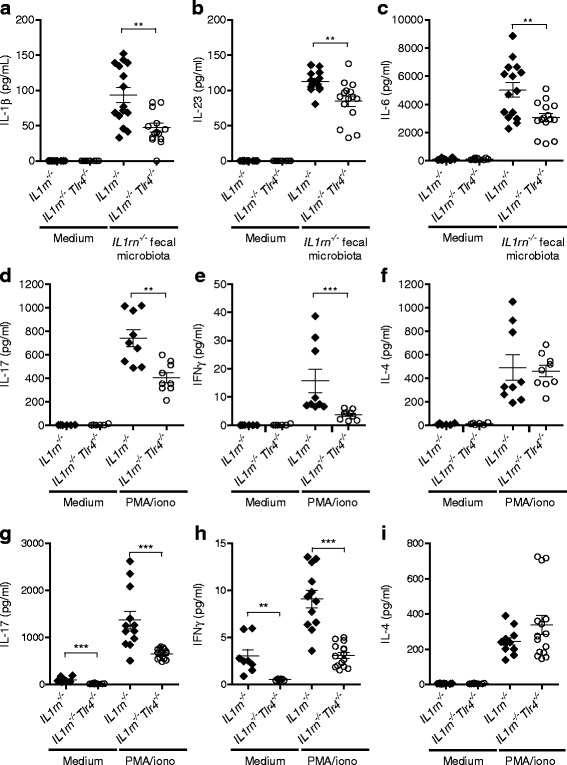
A significant role for TLR4 in intestinal production of cytokines involved in LP Th17 differentiation. **a**–**c** Production of IL-1β, IL-23, and IL-6 by SI-LP mononuclear cells of *IL1rn* ^−/−^ and *IL1rn* ^−/−^ *Tlr4* ^−/−^ mice cultured in the presence of autoclaved *IL1rn* ^−/−^ complete fecal microbial antigens for 24 h. **d**–**f** Cytokine production by SI-LP lymphocytes of separately housed *IL1rn* ^−/−^ and *IL1rn* ^−/−^ *Tlr4* ^−/−^ mice ex vivo stimulated with PMA and ionomycin for 5 h. **g**–**i** Cytokine production by SI-LP lymphocytes of *IL1rn* ^−/−^ and *IL1rn* ^−/−^ *Tlr4* ^−/−^ mice co-housed for 10 days. Cells were stimulated as in **d**–**f**. *P ≤ 0.05, **P ≤ 0.01, ***P ≤ 0.001, by Mann-Whitney *U* test

We next examined the role of TLR4 in the mucosal T cell response in *IL1rn* ^−/−^ mice. Th17 cells require transforming growth factor-β and IL-6, plus IL-1β in mouse, for initial differentiation, and IL-23 for their functional maturation and pathogenic function [24, 34]. To determine the role of TLR4 in response to *IL1rn* ^−/−^ intestinal microbial antigens, we cultured SI-LP mononuclear cells from *IL1rn* ^−/−^ and *IL1rn* ^−/−^ *Tlr4* ^−/−^ mice ex vivo with autoclaved *IL1rn* ^−/−^ intestinal microbiota. SI-LP mononuclear cells from *IL1rn* ^−/−^ *Tlr4* ^−/−^ mice produced significantly less IL-1β (Fig. 7a, *P* = 0.0042). Furthermore, the induction of IL-23 and IL-6 by *IL1rn* ^−/−^ fecal microbiota was partly TLR4-dependent (Fig. 7b, c, *P* = 0.0014 and *P* = 0.009, respectively). Reduced cytokine production in *IL1rn* ^−/−^ *Tlr4* ^−/−^ mice was not due to an altered composition of mononuclear cells in the SI-LP, because the percentage and abundance of CD11c^+^ MHCII^+^ DCs as well as distinct subsets of CD103^+^ CD11b^+^, CD103^+^ CD11b^−^, and CD11b^+^ CD103^−^ phagocytes were similar between *IL1rn* ^−/−^ and *IL1rn* ^−/−^ *Tlr4* ^−/−^ mice (data not shown). Importantly, stimulation of *IL1rn* ^−/−^ LP mononuclear cells with *IL1rn* ^−/−^ and *IL1rn* ^−/−^ *Tlr4* ^−/−^ fecal microbial antigens induced similar concentrations of IL-1β, IL-23, and IL-6 (Additional file 1: Figure S8). This suggests that the altered composition of microbiota in *IL1rn* ^−/−^ *Tlr4* ^−/−^ mice as such is not responsible for the lower production of these cytokines. These observations imply a significant role for TLR4 in intestinal production of the cytokines involved in LP Th17 differentiation in *IL1rn* ^−/−^ mice.

When cultured ex vivo with PMA and ionomycin, SI-LP cells from *IL1rn* ^−/−^ *Tlr4* ^−/−^ mice produced significantly less IL-17 compared with cells from *IL1rn* ^−/−^ *Tlr4* ^*+/+*^ mice before the onset of arthritis (*P* = 0.0028; Fig. 7d). The amount of IFNγ produced in this culture was about 50 folds lower than IL-17 and was significantly reduced in *IL1rn* ^−/−^ *Tlr4* ^−/−^ mice as well (Fig. 7e). However, IL-4 levels remained unaffected (Fig. 7f). LP T cells from *IL1rn* ^−/−^ *Tlr4* ^−/−^ mice still produced significantly less IL-17 and IFNγ when these mice were co-housed with *IL1rn* ^−/−^ (*Tlr4* ^*+/+*^) mice to transfer the microbiota (Fig. 7g–i). Therefore, reduced LP IL-17 production in *IL1rn* ^−/−^ *Tlr4* ^−/−^ mice is a result of the difference in host TLR4 expression rather than altered microbiota in *IL1rn* ^−/−^ *Tlr4* ^−/−^ versus *IL1rn* ^−/−^ mice. Stimulation of LP mononuclear cells of these co-housed mice with fecal microbial antigens confirmed that cells from *IL1rn* ^−/−^ *Tlr4* ^−/−^ mice produce lower amounts of IL-1β, IL-23, and IL-6 regardless of stimulation with *IL1rn* ^−/−^ or *IL1rn* ^−/−^ *Tlr4* ^−/−^ microbiota (Additional file 1: Figure S9). These data suggest that TLR4 activation contributes to intestinal LP production of IFNγ and most notably IL-17, and these effects precede the onset of arthritis.

Importantly, production of IL-17 but not IFNγ in lymph nodes draining the inflamed joints was diminished in *IL1rn* ^−/−^ *Tlr4* ^−/−^ mice compared with *IL1rn* ^−/−^ mice (Additional file 1: Figure S10). This is in agreement with our previous study showing that TLR4 induces systemic and local IL-17 production and promotes arthritis in *IL1rn* ^−/−^ mice [12]. Together, these observations suggest an essential role for TLR4 in the induction of intestinal LP IL-17 production associated with extra-intestinal IL-17 levels and the development of arthritis in *IL1rn* ^−/−^ mice.

## Discussion

The intestinal microbiome has emerged as a key determinant of health and disease. Although advanced sequencing techniques have enabled microbiome profiling in rheumatic patients, study of the underlying mucosal responses and the functional impact on arthritis is limited in human subjects due to the requirement of invasive techniques. The animal studies presented here demonstrate aberrations in intestinal microbiota in mice developing spontaneous autoimmune arthritis, introduce commensal tobramycin-sensitive microbiota as potential triggers for arthritis, and suggest a role for TLR4 activation in mucosal induction of inflammatory pathways including Th17 induction associated with arthritis.

Our study identifies loss of microbial diversity and specific taxonomic alterations in the microbiota of autoimmune-prone *IL1rn* ^−/−^ mice. Importantly, loss of intestinal microbial diversity and richness also coincides with human autoimmune diseases such as diabetes and rheumatoid and psoriatic arthritis [14, 16, 35]. Among the microbiota increased in *IL1rn* ^−/−^ mice, *Streptococcus* species are known inducers of chronic TLR-mediated arthritis in animal models when injected intra-articularly [36, 37]. Furthermore, a commensal *Helicobacter* (*H. hepaticus*) has been shown to induce IL-23 and mediate T cell-dependent gut inflammation in immunocompromised mice [38]. The decreased *Barnesiella* in *IL1rn* ^−/−^ mice is consistent with a previous study associating the abundance of *Barnesiella* with resistance to arthritis in HLA-DRB1*0402 mice [39]. A specific species of *Prevotella*, *P. copri*, is overrepresented in patients with new-onset RA [14] and was recently shown to increase colonic Th17 cells and promote arthritis in SKG mice after co-exposure to the fungal component zymosan [18]. On the other hand, *P. histicola* has been reported to suppress collagen-induced arthritis in transgenic mice expressing RA-susceptibility gene HLA-DQ8 [40]. Therefore, the immunomodulatory effects of the gut microbiota, including *Prevotella*, are species- and sometimes even strain-dependent. Due to inherently limited resolution of 16S rRNA gene sequencing, our data on the abundance of *Prevotella* is limited to the genus level and the exact *Prevotella* species altered in *IL1rn* ^−/−^ mice remain unclear. Overall, it is tempting to speculate that complex alterations in several taxa determine the net mucosal response to affect arthritis. It should also be noted that fecal bacterial community structures do not fully mirror the site-specific luminal or mucosa-associated microbiota profiles and were used in this study as a proxy of the gut microbiota of the *Il1rn* ^−/−^ mice.

Our previous studies showed that *IL1rn* ^−/−^ *Tlr2* ^−/−^ mice develop a more severe arthritis compared with *IL1rn* ^−/−^ mice [12]. Given that additional TLR2 deficiency did not affect the microbiota of *IL1rn* ^−/−^ mice to a major extent (Fig. 1), we speculate that severe arthritis in *IL1rn* ^−/−^ *Tlr2* ^−/−^ mice is due to the altered host immune response, specifically reduced function of Treg cells [12], rather than alteration in the microbiome. However, the data regarding lack of a major influence of TLR2 deficiency on *Il1rn* ^−/−^ microbiota should be interpreted with caution due to the absence of littermate *Il1rn* ^−/−^ *Tlr2* ^*+/+*^ mice in our studies.

*IL1rn* ^−/−^ mice had specific expansion of intestinal Th17 cells. The pathogenic relevance of IL-17 in the development of arthritis in *IL1rn* ^−/−^ mice has been demonstrated before, since both IL-17 gene deficiency and treatment with neutralizing anti-IL-17 antibodies inhibit arthritis [4, 8]. A previous study showed that γδ T cells rather than Th17 cells represent most IL-17-producing T cells in the inflamed joints of *IL1rn* ^−/−^ mice [41]. While γδ17 and Th17 cells may have complementary pathogenic roles in the development of *IL1rn* ^−/−^ arthritis, our data suggest that IL-17-producing cells located in lamina propria and induced by *IL1rn* ^−/−^ intestinal microbiota are TCRβ-expressing CD4^+^ Th17 cells (Fig. 4 and Additional file 1: Figure S4).

The expansion of LP Th17 cells in *IL1rn* ^−/−^ mice was caused by the dysregulated microbiota as confirmed by fecal transfer experiments (Fig. 3g–i). A critical pathogenic link to the spontaneous arthritis was revealed by our germ-free and antibiotic treatment studies (Figs. 4 and 5). Other previous studies which demonstrated the involvement of the gut microbiota in exacerbation of autoimmune arthritis found SFB as the responsible microorganisms. One study showed a role for vancomycin-sensitive microbiota including SFB in the induction of IL-17- and autoantibody-driven arthritis in K/BxN mice [13], and another showed that SFB can lower the activation threshold of self-reactive T cells and promote the differentiation of arthritogenic Th1 cells in a T cell transfer model of arthritis [42]. Our data are the first to demonstrate that although SFB colonization exacerbates arthritis, among the dysregulated indigenous microbiota present in the *IL1rn* ^−/−^ mice, those sensitive to tobramycin, i.e.*, Helicobacter*, *Flexispira*, *Clostridium* and *Dehalobacterium*, are potential candidates to promote arthritis in a genetically susceptible host. This is relevant for human disease, given that SFB were not found in genome-wide sequences of 263 gut metagenomes of human adults [30, 31].

Our data also provide the first evidence for the involvement of TLR4 in defining the intestinal mucosal T cell phenotype. TLR4 activation of LP mononuclear cells by *IL1rn* ^−/−^ microbiota-induced IL-1β, IL-23 and IL-6 (Fig. 7a–c). Microbiota-induced IL-1β is critical for the development of steady-state Th17 cells in the gut [43]. IL-1 also synergizes with IL-6 and IL-23 to regulate early differentiation of Th17 cells and maintain cytokine expression in effector Th17 cells [44]. It was recently shown that infectious triggers such as influenza lung infection and colitis trigger an IL-1β-induced Th17 differentiation and promote arthritis induced by KRN transgenic T cells [45]. Interestingly, a subset of human CD14^+^ CD163^low^ lamina propria cells expressing both macrophage and DC markers has been found to express TLR4, produce IL-1β and IL-6 upon TLR4 stimulation, and induce Th17 differentiation [46]. However, the specific subset of LP phagocytes that orchestrates the phase-dependent TLR4-mediated mucosal response to microbiota in our studies remains to be determined.

Several studies have shown that TLR4 deficiency and systemic inhibition of TLR4 using specific antagonists or neutralizing antibodies can suppress experimental arthritis [12, 47–49]. Importantly, TLR4 is believed to be hyper-responsive in both blood monocyte-derived DCs and CD14^+^ synovial fluid macrophages of RA patients compared with healthy controls [50, 51]. Pathways associated with TLR signaling are upregulated in synovial fluid macrophages of patients with RA. A proinflammatory role for TLR4 during arthritis has previously been widely attributed to TLR4 activation by endogenous damage-associated molecular patterns present in the joint rather than microbial agonists [12, 49, 52, 53]. Our observations suggest that TLR4-mediated modulation of the mucosal immune response in intestinal LP may be another function involving TLR4 in arthritis.

## Conclusions

Our study reveals a crucial role for IL-1Ra in regulation of the diversity and the composition of intestinal microbiota and a balanced T cell response in the intestinal LP. We show that the aberrant microbiota in *IL1rn* ^−/−^ mice have the capacity to enhance LP Th17 cells which are associated with arthritis, likely via TLR4-induced production of IL-1β, IL-6, and IL-23. Although *IL1rn* ^−/−^ intestinal microbiota do not cause arthritis in a normal (WT) host, these microbiota, in particular tobramycin-sensitive bacteria, contribute to the development of arthritis in *IL1rn* ^−/−^ mice. Our data suggest that the interplay between IL-1Ra, intestinal microbiota, TLR4, and mucosal T cells may serve as a potential predisposing or initiating event in the context of autoimmune disease and provide opportunities to control RA.

## Methods

### Mice

*IL1rn* ^−/−^ mice on a BALB/c background were kindly provided by Dr. M. Nicklin (Sheffield, UK) [54]. WT BALB/c mice were purchased from Harlan, UK. Mice were co-housed in filter-top non-individually ventilated (non-IVC) cages in the same room in our animal facility for at least 8 weeks prior to feces collection for pyrosequencing. *IL1rn* ^−/−^ *Tlr4* ^−/−^ mice and their *IL1rn* ^−/−^ *Tlr* ^+/+^ littermates were generated as described before [12] and used for microbiota sequencing. *IL1rn* ^−/−^ *Tlr2* ^−/−^ mice were compared to non-littermate *IL1rn* ^−/−^ mice in this study.

### Microbiota sequencing and data analysis

Fecal bacterial DNA from 15-week-old mice was isolated using phenol-, chloroform-, isoamyl alcohol-based extraction (Sigma). Sequencing was performed by DNAVision (Charleroi, Belgium) on a Roche 454 GS-FLX System using 16S rRNA gene bar-coded primers targeting the V5-V6 conserved DNA regions (forward primer 784F: 5′-AGGATTAGATACCCTGGTA-3′, reverse primer 1061R: 5′-CRRCACGAGCTGACGAC-3′) [55]. For gene sequence analysis, a customized workflow based on Quantitative Insights Into Microbial Ecology (QIIME version 1.2) was adopted (http://qiime.org/) [56]. Settings recommended in QIIME 1.2 tutorial were applied. Additionally, reads were filtered for chimeric sequences using Chimera Slayer as described before [57]. OTU clustering was performed with settings as recommended by QIIME [58] using an identity threshold of 97%. The Ribosomal Database Project classifier version 2.2 was used for taxonomic classification [59]. Hierarchical clustering of samples was performed using UPGMA with weighted UniFrac as a distance measure as implemented in QIIME 1.2. For statistical analysis and generation of figures, a custom QIIME implemented R-package, SciPy [60] (www.Scipy.org), Graphpad Prism version 5.0, and Microsoft® Office Excel® 2007 were adopted. Presence of SFB was assessed by real-time quantitative PCR (qPCR) on fecal DNA using SFB-specific primers as described before [61]. The delta Ct (cycle threshold) value was calculated for SFB-specific rRNA gene relative to the total (conserved) bacterial 16S rRNA genes amplified using universal bacterial primers to correct for the total bacterial DNA input. Data are presented as delta Ct (ΔCt) and relative SFB expression calculated as 2^−ΔCt^ × 10,000 (Additional file 1: Table S3).

### Microbiota transfer and co-housing

*IL1rn* ^−/−^ microbiota were transferred to WT mice by oral gavage of 200 μl of a homogenized *IL1rn* ^−/−^ fecal suspension prepared in sterile PBS. Immediately hereafter, the gavaged WT mice were co-housed with *IL1rn* ^−/−^ mice in the same individually ventilated cage for a period of 6 weeks to ensure sustained microbiota transfer by coprophagy. The control WT mice were gavaged with their own fecal suspension and housed separate from *IL1rn* ^−/−^ fecal-transplanted mice in IVC cages. To verify microbiota of conventional *IL1rn* ^−/−^ mice can trigger arthritis, GF *IL1rn* ^−/−^ mice received either 200 μl of sterile water or 200 μl fecal suspensions of conventional *IL1rn* ^−/−^ mice and were monitored for the development of arthritis for 8 weeks. In some studies, *IL1rn* ^−/−^ mice were co-housed with *IL1rn* ^−/−^ *Tlr4* ^−/−^ mice for 10 days before analysis of LP T cells.

### Antibiotic treatments and reconstitution with SFB

Intestinal microbiota were depleted using a cocktail of metronidazole (Acros Organics), neomycin trisulfate (Sigma), and ampicillin sodium salt (Sigma) (all 1 g/l) provided in drinking water for 1 week. Indicated groups received 200 μl fecal suspensions of SFB-monocolonized mice by oral gavage 1 week after ceasing antibiotics. For single antibiotic treatments, ampicillin sodium salt (1 g/l), metronidazole (1 g/l), vancomycin hydrochloride (0.5 g/l, Fisher Scientific), or tobramycin sulfate (1 g/l, Centrafarm) was added to drinking water for 8 weeks and refreshed once a week. Sucrose (6 g/l) was added to drinking water of all groups including controls during treatments.

### Isolation of lamina propria cells

Lamina propria mononuclear cells were isolated from the small intestine and colon after removing mesenteric fat and Peyer’s patches, followed by incubation with 33 mM EDTA on ice for 30 min to remove epithelial cells, and subsequent digestion with 1 mg/ml collagenase-D (Roche) and 10 μg/ml DNAse I (Sigma) at 37 °C for three cycles of 15 min. LP lymphocytes were then harvested at the interphase of a 40:80% percoll gradient (Sigma), washed thoroughly, and used in culture as described below.

### Cell cultures and cytokine measurements

SI-LP mononuclear cells (4 × 10^5^ cells/well), LN cells (2 × 10^5^ cells/well), and splenocytes (1 × 10^5^ cells/well) were cultured in round-bottom 96-well plates in the presence of PMA (50 ng/ml; Sigma) and ionomycin (1 μg/ml; Sigma) for 5 or 6 h, as indicated in the figure legends. SI-LP cells were also cultured for 24 h in the presence of *IL1rn* ^−/−^ or *IL1rn* ^−/−^ *Tlr4* ^−/−^ complete microbial antigens (1:200 *v*/*v* ratio) prepared by autoclaving the *IL1rn* ^−/−^ fecal pellets dissolved in PBS, and then centrifuging the suspension at 2000 rpm for 5 min. Cytokine levels in culture supernatants were measured by Luminex using the mouse cytokine/chemokine magnetic bead kit (Milliplex and Bio-Rad).

### Flow cytometry

For intracellular cytokine staining, SI-LP cells were incubated with PMA (50 ng/ml; Sigma), ionomycin (1 μg/ml; Sigma), and Brefeldin A (1 μl/ml; BD Biosciences) at 37 °C for 4 h. Cells were stained with fixable viability dye Efluor780 (eBioscience), anti-TCRβ-FITC (Biolegend) or anti-CD3-PE (BD pharmigen), and anti-CD4-APC (Biolegend), then fixed and permeabilized using fixation/permeabilization buffer (eBioscience), and stained with anti-IL-17-FITC (Biolegend), IL-17-PECy7 (Biolegend), or anti-IFNγ-FITC (BD pharmingen) in permeabilization buffer (eBioscience).

### Assessment of arthritis

Severity of arthritis was scored using a previously standardized arbitrary scoring system on a 0–2 scale per paw [12]. Arthritis developed only in ankle joints (maximum score of 4). For histology, total ankle joints were isolated and fixed in 4% formaldehyde for 4 days, thereafter decalcified in 5% formic acid and embedded in paraffin. Tissue sections of 7 μm were stained using hematoxylin and eosin to study synovial inflammation, cartilage destruction, and bone erosion. Each parameter was scored on a scale from 0 to 3 in a blinded manner.

### Statistics

Group measures are expressed as mean + SEM. Statistical significance was tested using an unpaired two-tailed Mann-Whitney *U* test to compare two and ANOVA to compare more groups, with Bonferroni correction for multiple testing when applicable (GraphPad Prism 5.0). Arthritis scores were compared using repeated measures ANOVA with Bonferroni correction. Significance is indicated on figures as follows: n.s. (not significant), *P < 0.05, **P < 0.01, ***P < 0.001.

## Additional file

Additional file 1: Figure S1. Hierarchical clustering of wild-type and *IL1rn* ^−/−^ mice based on intestinal microbiota. **Figure S2.** The impact of lineage origin versus IL1rn-deficiency on the overall fecal microbiota composition. **Figure S3.** Gating strategy. **Figure S4.** Frequencies and numbers of IL-17-producing cells among TCRβ^+^ and TCRβ^−^ T cell populations with and without CD4 expression. **Figure S5.** Colonization of germ-free (GF) *IL1rn* ^−/−^ mice with fecal microbiota of conventional *IL1rn* ^−/−^ mice increases the severity of arthritis. **Figure S6.** Increased expression of Th2/Treg cytokines in the spleens but not in the popliteal lymph nodes (LN) of germ-free *IL1rn* ^−/−^ mice. **Figure S7.** Effects of 8-week oral tobramycin treatment on microbiota of *IL1rn* ^−/−^ mice assessed by 16S gene sequencing of fecal bacterial DNA. **Figure S8.** *IL1rn* ^−/−^ and *IL1rn* ^−/−^ *Tlr4* ^−/−^ microbiota induce similar cytokine response in lamina propria mononuclear cells. **Figure S9.** Lamina propria mononuclear cells of *IL1rn* ^−/−^ *Tlr4* ^−/−^ mice co-housed with *IL1rn* ^−/−^ mice produce less Th17-inducing cytokines. **Figure S10.** Decreased IL-17 production in draining lymph nodes of TLR4 deficient mice. **Table 1.** The average and total number of (assigned) reads and operational taxonomic units (OTU) per experimental group. **Table 2.** TLR4 deficiency normalizes specific aberrations in *Il1rn* ^−/−^ intestinal microbiome toward WT level. **Table 3.** Assessment of the presence of SFB expression in WT, *IL1rn* ^−/−^, *IL1rn* ^−/−^ *Tlr2* ^−/−^, and *IL1rn* ^−/−^ *Tlr4* ^−/−^ mice. **Table 4.** Alterations in fecal microbiota  by oral tobramycin. (DOCX 1237 kb)
