# Supplementary material for: Aberrant intestinal microbiota due to IL-1 receptor antagonist deficiency promotes IL-17- and TLR4-dependent arthritis
Source: Microbiome. 2017 Jun 23;5:63. doi: 10.1186/s40168-017-0278-2 (PMC5481968; doi:10.1186/s40168-017-0278-2)
Supplement: Additional file 1: Figure S1. — Hierarchical clustering of wild-type and IL1rn −/− mice based on intestinal microbiota. Figure S2. The impact of lineage origin versus IL1rn-deficiency on the overall fecal microbiota composition. Figure S3. Gating strategy. Figure S4. Frequencies and numbers of IL-17-producing cells among TCRβ+ and TCRβ− T cell populations with and without CD4 expression. Figure S5. Colonization of germ-free (GF) IL1rn −/− mice with fecal microbiota of conventional IL1rn −/− mice increases the severity of arthritis. Figure S6. Increased expression of Th2/Treg cytokines in the spleens but not in the popliteal lymph nodes (LN) of germ-free IL1rn −/− mice. Figure S7. Effects of 8-week oral tobramycin treatment on microbiota of IL1rn −/− mice assessed by 16S gene sequencing of fecal bacterial DNA. Figure S8. IL1rn −/− and IL1rn −/− Tlr4 −/− microbiota induce similar cytokine response in lamina propria mononuclear cells. Figure S9. Lamina propria mononuclear cells of IL1rn −/− Tlr4 −/− mice co-housed with IL1rn −/− mice produce less Th17-inducing cytokines. Figure S10. Decreased IL-17 production in draining lymph nodes of TLR4 deficient mice. Table 1. The average and total number of (assigned) reads and operational taxonomic units (OTU) per experimental group. Table 2. TLR4 deficiency normalizes specific aberrations in Il1rn −/− intestinal microbiome toward WT level. Table 3. Assessment of the presence of SFB expression in WT, IL1rn −/−, IL1rn −/− Tlr2 −/−, and IL1rn −/− Tlr4 −/− mice. Table 4. Alterations in fecal microbiota by oral tobramycin. (DOCX 1237 kb) [file 40168_2017_278_MOESM1_ESM.docx]

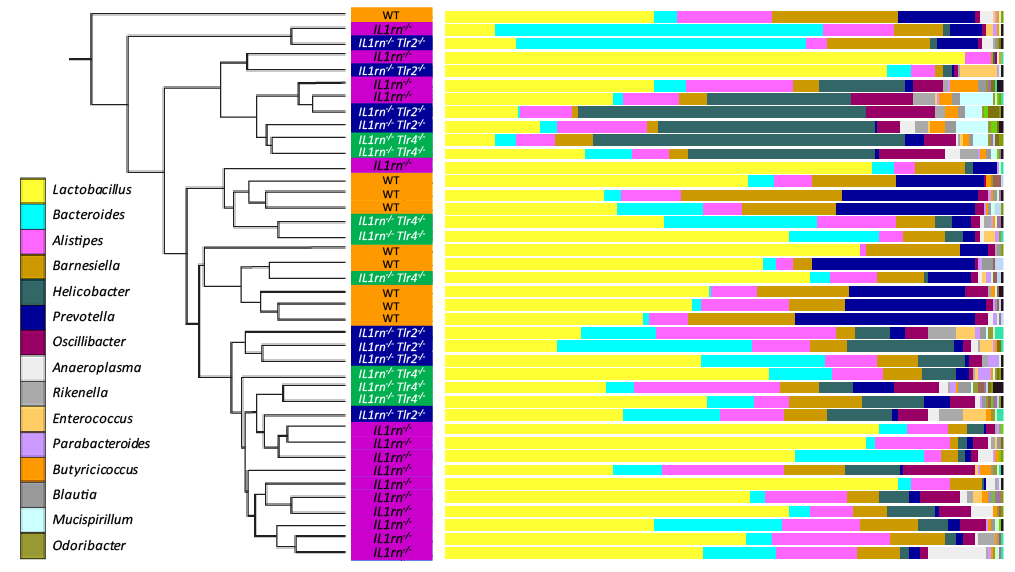


**Supplementary Figure 1. Hierarchical clustering of wild-type and *IL1rn^-/-^* mice based on intestinal microbiota.** Hierarchical weighed UniFrac clustering of fecal samples of WT (n=9), *IL1rn^-/-^* (n=15), *IL1rn^-/-^ Tlr2^-/-^* (n=8) and *IL1rn^-/-^ Tlr4^-/-^* (n=8) mice based on their intestinal microbial composition. Data were obtained from 16S rRNA gene 454-pyrosequencing of fecal bacterial DNA of 15-week-old mice. The dashed red lines highlight the mice clustered together based on their intestinal microbiota.

**Supplementary Figure 2.** The impact of lineage origin (A) versus *IL1rn*-deficiency (B) on the overall fecal microbiota composition. (A) To assess the effects of lineage origin and caging, the weighted UniFrac distance was calculated for each mouse in a colony versus all other mice of the same genotype in the same litter (intra-cage distances) and all other mice of the same genotype from a different litter (inter-cage effects). (B) The effect of genotype (WT or *IL1rn^-/-^*) is shown as the weighted UniFrac distance for each mouse versus all other mice from either the same or the opposite genotype. A higher UniFrac distance indicates greater dissimilarity between the microbial communities. Error bars indicate mean ± SEM. n.s. not significant; *P ≤ 0.05, **P ≤ 0.01, and *** P ≤ 0.001 by Mann-Whitney U test.

**
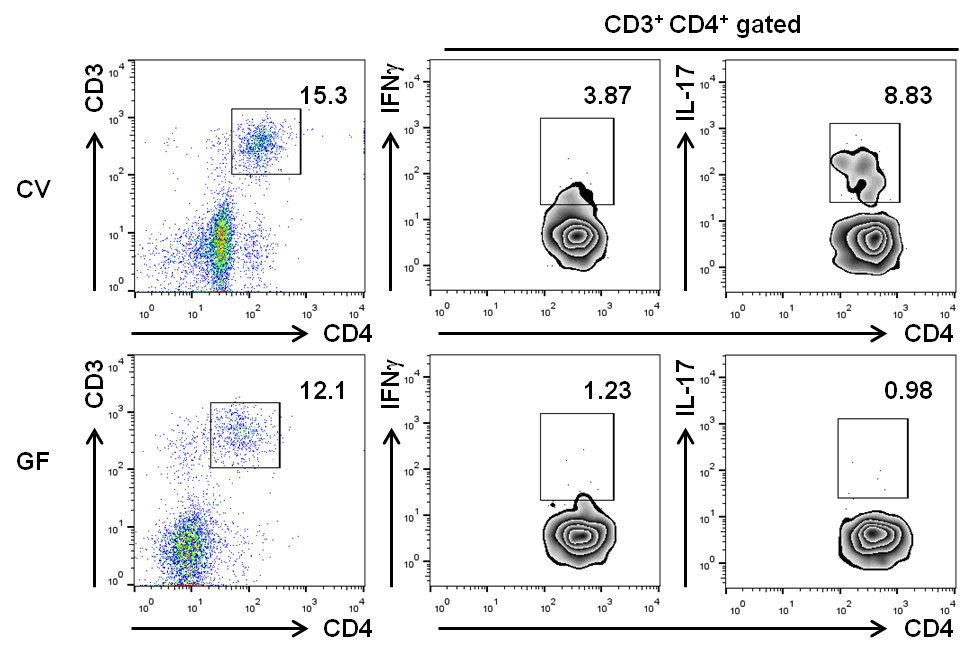
**

**Supplementary Figure 3. Gating strategy.** Flow cytometry gating strategy used to identify IFNγ and IL-17 producing CD3^+^CD4^+^ T cells in small intestine lamina propria of conventional (CV) and germ-free (GF) *IL1rn*^-/-^ mice.

**
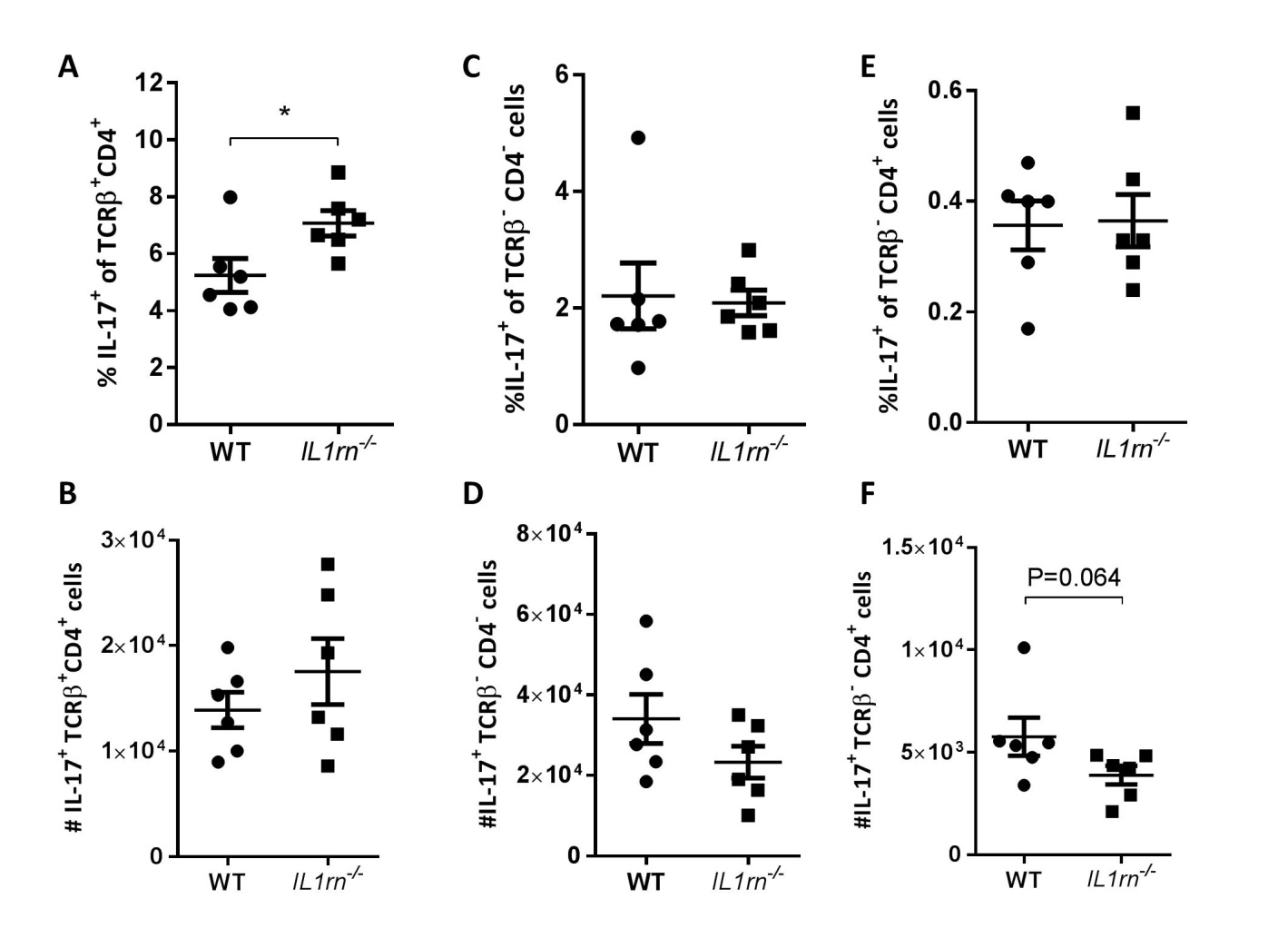
**

**Supplementary Figure 4. Frequencies and numbers of IL-17-producing cells among TCRβ^+^ and TCRβ^-^ T cell populations with and without CD4 expression.** Only the proportion of TCRβ^+^ CD4^+^ IL-17^+^ (Th17) cells shows significant increase in lamina propria of *IL1rn^-/-^* mice. Error bars indicate mean ± SEM. *P ≤ 0.05 by Mann-Whitney U test.

**Supplementary Figure 5. Colonization of germ-free (GF) *IL1rn*^-/-^ mice with fecal microbiota of conventional *IL1rn*^-/-^ mice increases the severity of arthritis.** GF *IL1rn*^-/-^ mice received either 200 µl of sterile water or 200 µl fecal suspension of conventional *IL1rn*^-/-^  mice and were monitored for the development of arthritis for 8 weeks. *P ≤ 0.05.


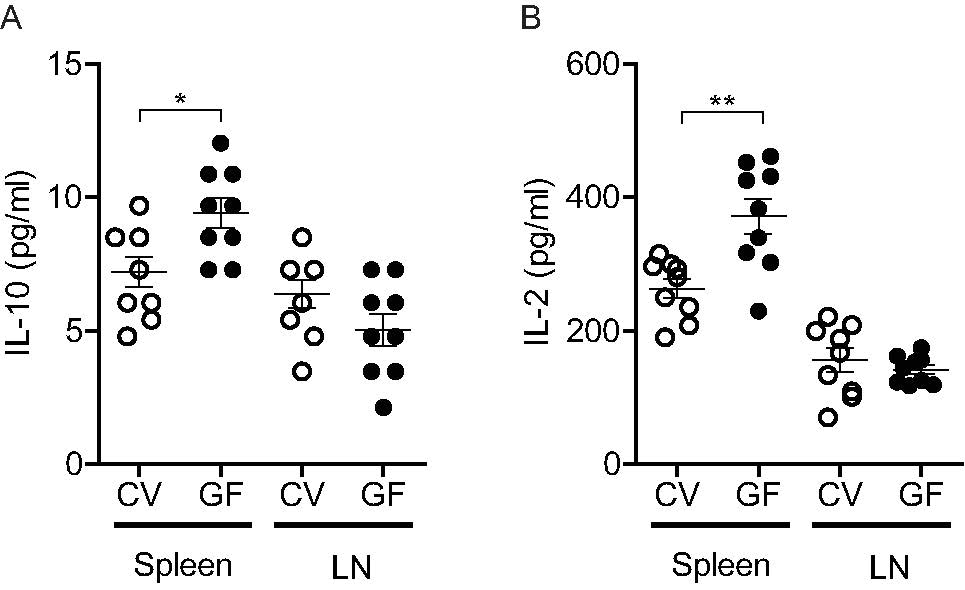


**Supplementary Figure 6.** **Increased expression of Th2/Treg cytokines in spleens but not popliteal lymph nodes (LN) of germ-free *IL1rn^-/-^* mice.** (A-B) Production of IL-10 and IL-2 upon *ex vivo* stimulation of spleen and lymph node cells from conventional (CV) and germ-free (GF) mice with PMA and ionomycin for 6 hours, as measured by Luminex assay. n=3 mice per group each stimulated in triplicate. n.s. not significant, *P ≤ 0.05 and **P ≤ 0.01, by Mann-Whitney U test.


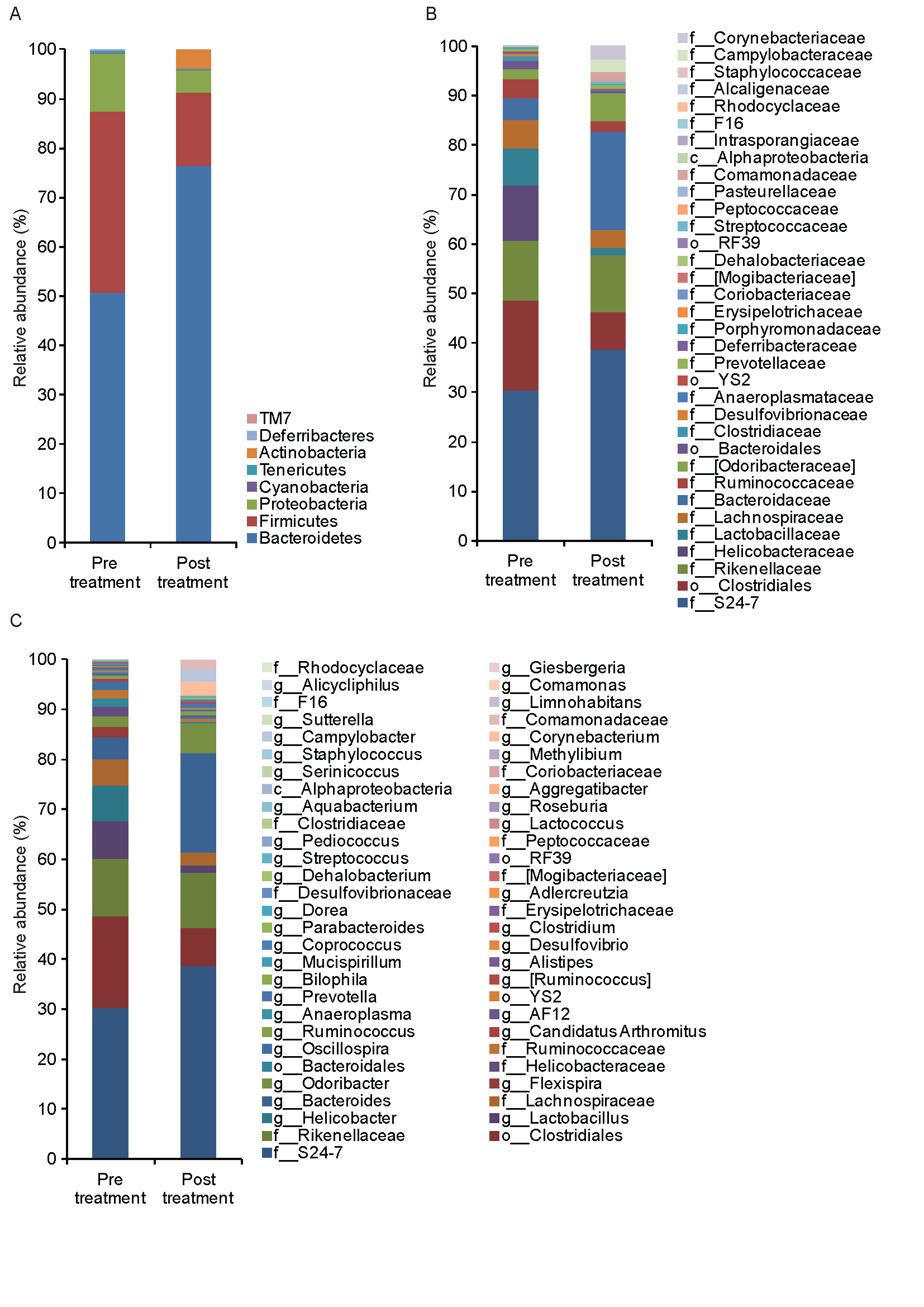


**Supplementary Figure 7. Effects of 8 weeks oral tobramycin treatment on microbiota of *IL1rn^-/-^* mice assessed by 16S gene sequencing of fecal bacterial DNA.** Fecal pellets were collected at the baseline and at the end-point (8 weeks) of treatment. o=order, f= family and g=genus.


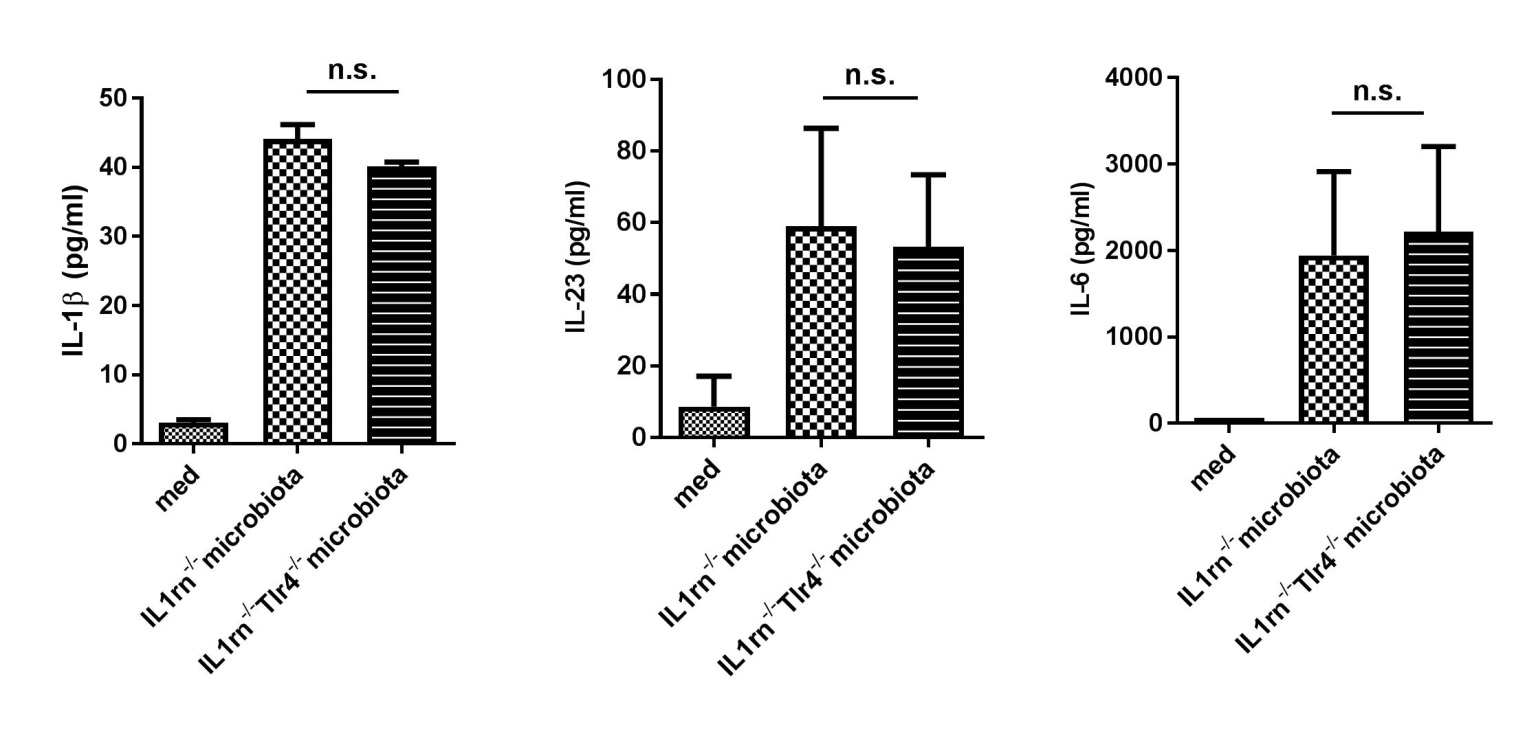
**Supplementary Figure 8. *IL1rn^-/-^* and *IL1rn^-/-^ Tlr4^-/-^* microbiota induce similar cytokine response in lamina propria mononuclear cells.** Production of IL-1β, IL-23 and IL-6 by small intestine lamina propria mononuclear cells of *IL1rn^-/-^* mice cultured in the presence of autoclaved *IL1rn^-/-^* and *IL1rn^-/-^ Tlr4^-/-^* complete fecal microbial antigens (1:200 v/v ratio) for 24 hours. n.s., not significant.


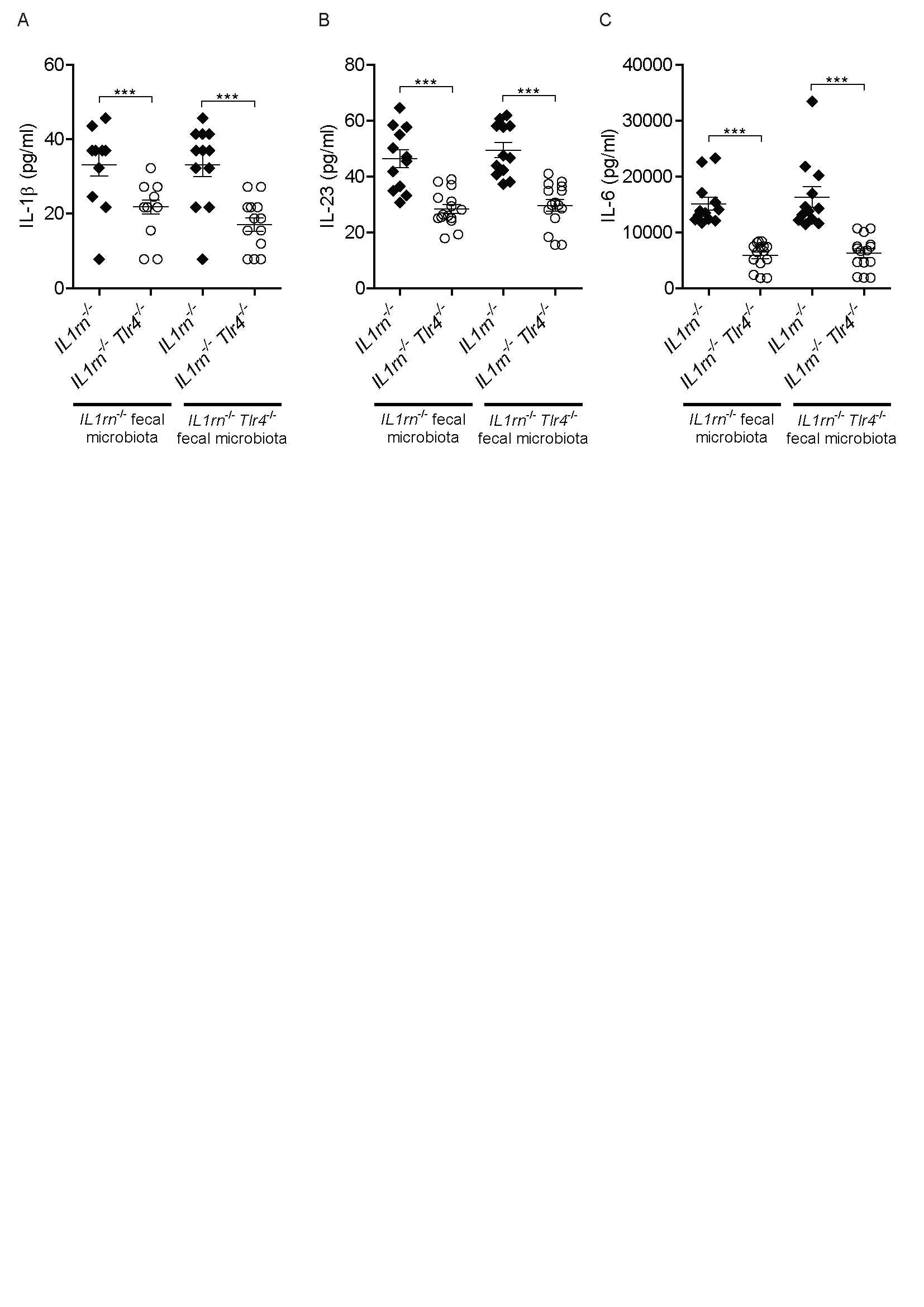


**Supplementary Figure 9**. **Lamina propria mononuclear cells of *IL1rn^-/-^* *Tlr4^-/-^* mice co-housed with *IL1rn^-/-^* mice produce less Th17-inducing cytokines.** Production of IL-1β, IL-23 and IL-6 by lamina propria mononuclear cells of *IL1rn^-/-^* and *IL1rn^-/-^* *Tlr4^-/-^* mice co-housed for 10 days. Cells were stimulated for 24 hours with fecal microbial antigens from (separately housed) *IL1rn^-/-^* and *IL1rn^-/-^* *Tlr4^-/-^* mice. ***P ≤ 0.001 by Mann-Whitney U test.


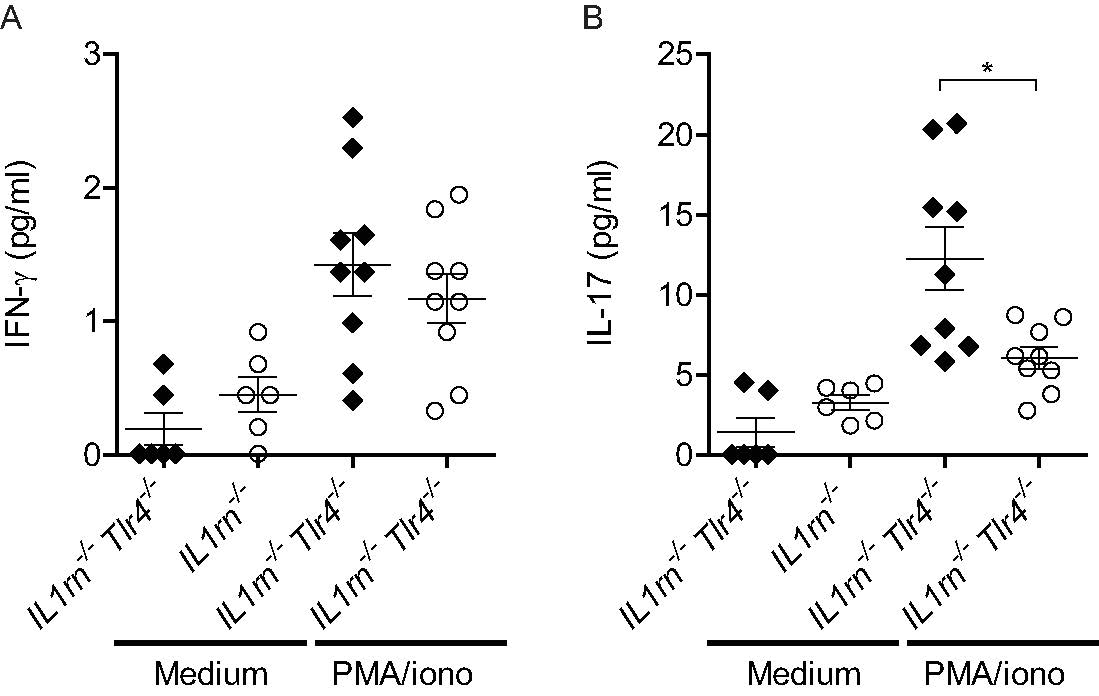


**Supplementary Figure 10. Decreased IL-17 production in draining lymph nodes of TLR4 deficient mice.** (A-B) Cytokine production by draining lymph node cells of *IL1rn^-/-^ and IL1rn^-/-^ Tlr4^-/-^* mice *ex vivo* stimulated with PMA and ionomycin for 5 hours.

**Supplementary Table 1**. The average and total number of (assigned) reads and operational taxonomic units (OTU) per experimental group. In addition, the number and percentage of reads assigned to phylum or genus level are shown.

|  | | **Reads** | | | **OTU** | | | **Assigned at Phylum** | | **Assigned at Genus** | |  |
| --- | --- | --- | --- | --- | --- | --- | --- | --- | --- | --- | --- | --- |
| ***Genotype*** |  | ***Average*** | ***SEM*** | ***Total*** | ***Average*** | ***SEM*** | ***Total*** | ***Total Reads*** | **%** | ***Total Reads*** | **%** | ***Group Size*** |
| Wild Type | | 4286 | 626 | 38576 | 617 | 57 | 5554 | 37890 | 98.2% | 15159 | 39.3% | n = 9 |
| *IL1rn^-/-^* | | 5947 | 462 | 89200 | 524 | 28 | 7854 | 88539 | 99.3% | 37284 | 41.8% | n = 15 |
| *IL1rn^-/-^ Tlr2^-/-^* | | 5897 | 432 | 47179 | 661 | 41 | 5291 | 46461 | 98.5% | 17338 | 36.7% | n = 8 |
| *IL1rn^-/-^ Tlr4^-/-^* | | 9364 | 936 | 74910 | 1120 | 74 | 8957 | 73565 | 98.2% | 23601 | 31.5% | n = 8 |

**Supplementary Table 2.** TLR4 deficiency normalizes specific aberrations in *Il1rn^-/-^* intestinal microbiome towards WT level. A full list of significantly altered microbial taxa in *IL1rn^-/-^* mice compared to WT controls and the taxa normalized in *IL1rn^-/-^ Tlr4^-/-^* mice. Significant alterations by Mann-Whitney U test are highlighted in light green and those significant after Bonferroni correction are highlighted in dark green.

**
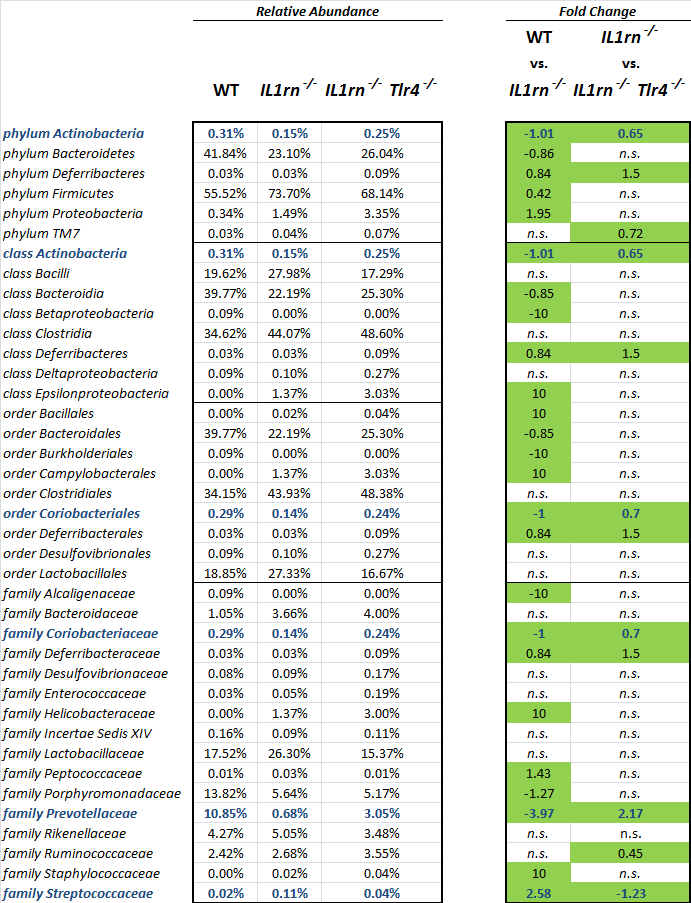
**

**
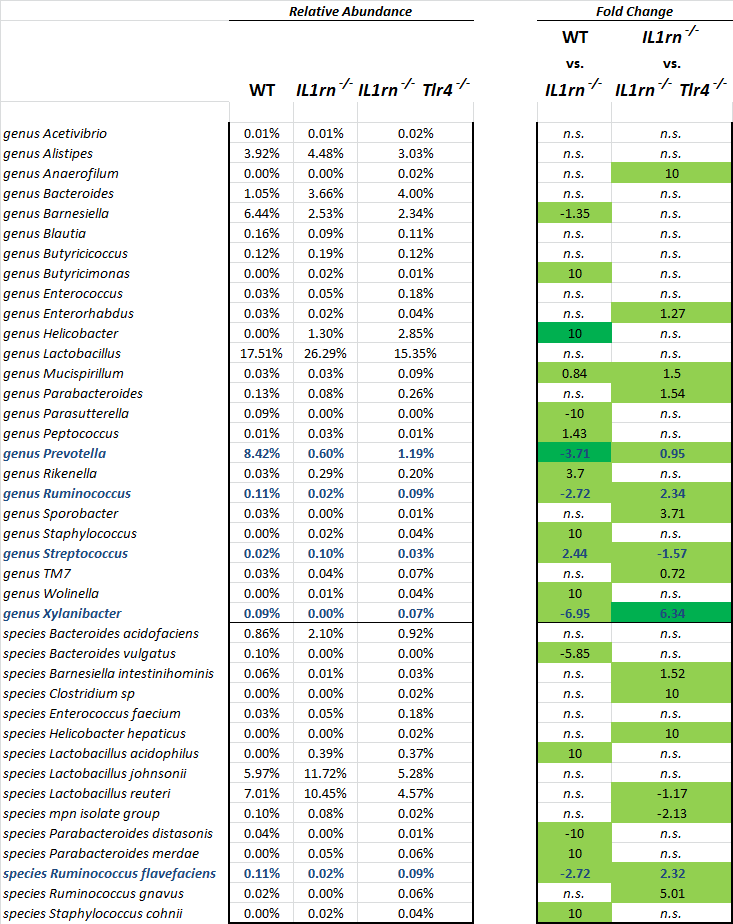
**

**Supplementary Table 3.** **Assessment of the presence of SFB expression in WT, *IL1rn*^-/-^, *IL1rn*^-/-^*Tlr2*^-/-^, *IL1rn*^-/-^*Tlr4*^-/-^ mice.** The Ct (cycle threshold) value for SFB-specific 16S rRNA gene by qPCR is shown. The delta Ct (ΔCt) value was calculated for SFB-specific 16S rRNA gene relative to the total (conserved) bacterial 16S rRNA genes amplified using universal bacterial primers. Data are presented as relative SFB expression calculated as 2^-ΔCt^ x 10,000. Mean ± SEM per experimental group is shown.

| **Genotype** | **Number of mice** | **number of mice with detectable SFB DNA by qPCR** | **Range of Ct value for SFB DNA by SFB-specific qPCR** | **ΔCt (Ct by SFB primers – Ct by universal primers)** | **Relative SFB DNA corrected for universal bacterial 16S DNA (2^-ΔCt^ x 10,000)** |
| --- | --- | --- | --- | --- | --- |
| **WT** | 9 | 9 | 31.2 - 36.3 | 15.49 ± 0.64 | 0.52 ± 0.23 |
| ***IL1rn^-/-^*** | 15 | 10 | 32.6 – undetectable | 18.29 ± 0.85 | 0.22 ± 0.14 |
| ***IL1rn^-/-^ Tlr2^-/-^*** | 8 | 6 | 28.4 – undetectable | 17.60 ± 2.01 | 0.31 ± 0.25 |
| ***IL1rn^-/-^ Tlr4^-/-^*** | 8 | 6 | 32.8 – undetectable | 19.79 ± 1.44 | 0.078 ± 0.047 |

**Supplementary Table 4. Alterations in fecal microbiota with a relative abundance > 0.1% by oral tobramycin, sorted by the abundance at the baseline.** Fecal DNA samples at baseline and at 8 weeks post-tobramycin treatment are compared. Significant alterations by Mann-Whitney U test are highlighted in green and those significant after Bonferroni correction for multiple testing are highlighted in blue. N=9 mice per group.

|  | Baseline | | Post-tobramycin treatment | |
| --- | --- | --- | --- | --- |
|  | **Mean** | **SEM** | **Mean** | **SEM** |
| family S24-7 | 30.367 | 5.990 | 38.709 | 6.606 |
| order Clostridiales | 18.373 | 4.275 | 7.707 | 1.731 |
| family Rikenellaceae | 11.375 | 1.275 | 11.001 | 2.964 |
| genus *Lactobacillus* | 7.596 | 1.892 | 1.402 | 0.572 |
| genus *Helicobacter* | 7.181 | 2.991 | 0.001 | 0.001 |
| family Lachnospiraceae | 5.313 | 0.997 | 2.595 | 1.042 |
| genus *Bacteroides* | 4.254 | 1.178 | 20.061 | 3.936 |
| genus *Flexispira* | 2.246 | 0.496 | 0.000 | 0.000 |
| genus *Odoribacter* | 2.105 | 0.375 | 5.750 | 2.088 |
| family Helicobacteraceae | 1.819 | 0.710 | 0.000 | 0.000 |
| order Bacteroidales | 1.717 | 0.549 | 0.458 | 0.141 |
| family Ruminococcaceae | 1.698 | 0.515 | 0.672 | 0.269 |
| genus *Oscillospira* | 1.645 | 0.457 | 0.611 | 0.147 |
| genus *Ruminococcus* | 0.539 | 0.153 | 0.757 | 0.228 |
| genus *AF12* | 0.419 | 0.065 | 0.297 | 0.121 |
| genus *Anaeroplasma* | 0.357 | 0.141 | 0.180 | 0.078 |
| order YS2 | 0.346 | 0.125 | 0.096 | 0.061 |
| genus *Prevotella* | 0.340 | 0.109 | 0.778 | 0.168 |
| genus [*Ruminococcus*] | 0.258 | 0.093 | 0.448 | 0.137 |
| genus *Bilophila* | 0.232 | 0.097 | 0.010 | 0.008 |
| genus *Alistipes* | 0.161 | 0.036 | 0.083 | 0.043 |
| genus *Mucispirillum* | 0.155 | 0.098 | 0.000 | 0.000 |
| genus *Desulfovibrio* | 0.120 | 0.053 | 0.007 | 0.005 |
| genus *Clostridium* | 0.107 | 0.036 | 0.000 | 0.000 |
| genus *Coprococcus* | 0.104 | 0.041 | 0.425 | 0.127 |
| genus *Parabacteroides* | 0.092 | 0.038 | 0.277 | 0.086 |
| family Erysipelotrichaceae | 0.072 | 0.029 | 0.038 | 0.020 |
| genus *Dorea* | 0.051 | 0.021 | 0.145 | 0.062 |
| genus *Dehalobacterium* | 0.049 | 0.015 | 0.000 | 0.000 |
| family [Mogibacteriaceae] | 0.043 | 0.013 | 0.000 | 0.000 |
| family Desulfovibrionaceae | 0.042 | 0.013 | 0.030 | 0.025 |
| genus *Adlercreutzia* | 0.042 | 0.019 | 0.000 | 0.000 |
| order RF39 | 0.023 | 0.023 | 0.000 | 0.000 |
| family Peptococcaceae | 0.017 | 0.005 | 0.000 | 0.000 |
| genus *Streptococcus* | 0.007 | 0.004 | 0.293 | 0.293 |
| genus *Aquabacterium* | 0.003 | 0.003 | 0.022 | 0.022 |
| genus *Corynebacterium* | 0.000 | 0.000 | 2.642 | 2.642 |
| genus *Campylobacter* | 0.000 | 0.000 | 2.452 | 2.452 |
| family Comamonadaceae | 0.000 | 0.000 | 1.952 | 1.952 |
| genus *Sutterella* | 0.000 | 0.000 | 0.024 | 0.018 |
| genus *Limnohabitans* | 0.000 | 0.000 | 0.018 | 0.018 |
